# TRAF4-mediated ubiquitination-dependent activation of JNK/Bcl-xL drives radioresistance

**DOI:** 10.1038/s41419-023-05637-y

**Published:** 2023-02-10

**Authors:** Xin Dong, Xiaoying Li, Yu Gan, Jie Ding, Baojun Wei, Li Zhou, Wei Cui, Wei Li

**Affiliations:** 1 Department of Clinical Laboratory, National Cancer Center/National Clinical Research Center for Cancer/Cancer Hospital, Chinese Academy of Medical Sciences and Peking Union Medical College, Beijing, 100021 China; 2 Department of Radiology, The Third Xiangya Hospital, Central South University, Changsha, Hunan 410013 China; 3 Department of Anesthesia, Fuwai Hospital, National Center for Cardiovascular Diseases, Chinese Academy of Medical Sciences and Peking Union Medical College, Beijing, 100000 China; 4 Department of Pathology, National Clinical Research Center for Geriatric Disorders, Xiangya Hospital of Central South University, Changsha, Hunan 410008 China

**Keywords:** Colorectal cancer, Cancer therapeutic resistance

## Abstract

The E3 ligase TNF receptor-associated factor 4 (TRAF4) is upregulated and closely associated with tumorigenesis and the progression of multiple human malignancies. However, its effect on radiosensitivity in colorectal cancer (CRC) has not been elucidated. The present study found that TRAF4 was significantly increased in CRC clinical tumor samples. Depletion of TRAF4 impaired the malignant phenotype of CRC cells and sensitized irradiation-induced cell death. Irradiation activated the c-Jun N-terminal kinases (JNKs)/c-Jun signaling via increasing JNKs K63-linked ubiquitination and phosphorylation. Furthermore, c-Jun activation triggered the transcription of the antiapoptotic protein Bcl-xL, thus contributing to the radioresistance of CRC cells. TRAF4 was positively correlated with c-Jun and Bcl-xL, and blocking TRAF4 or inhibiting Bcl-xL with inhibitor markedly promoted ionizing radiation (IR)-induced intrinsic apoptosis and sensitized CRC cells to radiotherapy in vitro and in vivo. Our findings illustrate a potential mechanism of radioresistance, emphasizing the clinical value of targeting the TRAF4/Bcl-xL axis in CRC therapy.

## Introduction

Colorectal cancer (CRC) has the fourth highest associated mortality worldwide, resulting in ~900,000 individuals succumbing to the disease yearly [1]. For patients with intermediate to advanced CRC who cannot tolerate surgery or chemotherapy, radiotherapy is an important treatment option in a clinical setting [2–4]. However, due to inter-individual differences in radiotherapy sensitivity, only a small percentage of patients respond well to radiotherapy [5, 6]. Therefore, it is essential to identify potential targets to improve the radiosensitivity of CRC.

Radiation therapy (RT) is the most effective cytotoxic treatment based on ionizing radiation (IR). It can trigger a variety of cell death, including apoptosis, necroptosis and autophagy-dependent cell death, to induce tumor cell death [7]. Among them, apoptosis is one of the most important. The modulation of apoptosis-related factors may enhance the sensitivity of tumors to radiation therapy and ultimately contribute to the effectiveness of cancer treatment. Tumor necrosis factor (TNF) receptor-associated factor 4 (TRAF4), a member of the TRAF family, is an E3 ubiquitin ligase that is mainly involved in cell proliferation, polarity, oxidation, apoptosis, immunity and other physiological processes [8]. It has been shown that TRAF4 is highly expressed in a variety of cancer types and promotes cancer cell proliferation, metastasis, and chemoresistant through signaling pathways such as NF-κB [9], AKT [10, 11], Wnt/β-catenin [12, 13] and CHK1 [14].

The present study found that TRAF4 was an essential E3 ligase associated with CRC radioresistance. The current findings demonstrated that depletion of TRAF4 downregulated the expression of Bcl-xL to enhance the IR-induced apoptosis of CRC cells in a JNK1/2-dependent manner. These results suggest that targeting the TRAF4/JNK/Bcl-xL axis could be a promising avenue to overcome the radioresistance of CRC.

## Materials and methods

### Reagents and antibodies

The chemicals, including z-VAD-fmk, necrostatin-1, 3-MA, A-1155463, cycloheximide (CHX) and MG132, were obtained from Selleck Chemicals (Houston, TX). RPMI-1640 and DMEM media, fetal bovine serum (FBS) and penicillin-streptomycin were acquired from Invitrogen (Thermo Fisher Scientific, Inc.). Control (Ctrl) small interfering RNA (siRNA) (#sc-93314), and siRNAs targeting Bcl-xL (#sc-77361), c-Jun (#sc-29223), p65 (#sc-29410), STAT5 (#sc-29495), and Ets1 (#sc-29309), were purchased from Santa Cruz Biotechnology, Inc. The transfection reagent Lipofectamine™ 2000 (#11668019) was purchased from Thermo Fisher Scientific, Inc. Antibodies against Bcl-xL (#2764, IB: 1:1000, IHC: 1:300), Mcl-1 (#5453, IB: 1:1000), Bcl-2 (#2870, IB: 1:1000), Ub-k48 (#8081, IB: 1:1000), Ub-k63 (#5621, IB: 1:1000), γ-H2AX (#9718, IB: 1:4000), cleaved-caspase 3 (#9661, IB: 1:1000), cleaved-PARP (#5625, IB: 1:1000), cytochrome C (#11940, IB: 1:1000), Bax (#14796, IB: 1:1000), β-actin (#3700, IB: 1:10,000), α-Tubulin (#2125, IB: 1:5000), c-Jun (#9165, IB: 1:1000, IHC: 1:300), p-c-Jun (#91952, IB: 1:1000), p-ERK1/2 (#4370, IB: 1:1000), p38 MAPK (#9212, IB: 1:1000), p-p38 MAPK (#4511, IB: 1:1000), JNK1/2 (#9252, IB: 1:1000), p-JNK1/2 (#9251, IB: 1:1000) NF-κB p65 (#8242, IB: 1:1000), STAT5 (#25656, IB: 1:1000), mouse IgG HRP (#7076, IB: 1:10,000) and rabbit IgG HRP (#7074, IB: 1:10000) were purchased from Cell Signaling Technology, Inc. Flag tag (#F3165, IB: 1:10000) and TRAF4 (#MABC985, IB: 1:4000, IHC: 1:300) were obtained from Sigma-Aldrich (Merck KGaA). Anti-Ets1 antibody (#sc-350, IB: 1:1000) was purchased from Santa Cruz Biotechnology, Inc. while anti-Ki-67 antibody (#ab15580, IHC: 1:300) was purchased from Abcam.

### Cell lines and cell culture

Normal human colonic epithelial cells FHC and colorectal cancer cells HCT116 and HT29, and 293 T cells were purchased from American Type Culture Collection (ATCC, Manassas, VA), and cultured according to the manufacturer’s protocols. All cells were incubated in a humidified incubator (37 ˚C, 5% CO_2_) and tested for mycoplasma every 2 months. The radioresistant cell lines HCT116R and HT29R were established in our laboratory as previously described [15].

### Cell viability assay

The viability of CRC cells was determined by MTS assay. Briefly, HCT116 and HT29 cells (2 × 10^3^ cells/well) were seeded into a 96-well plate and treated with or without IR (4 Gy). After incubation for 48 h at a 37˚C, MTS regent (#G3581, Promega, Madison, WI) was added to each well and cultured for another 1 h. Cell viability was measured following the standard protocol.

### Plate colony formation assay

CRC cells were treated with or without IR (4 Gy) and seeded at 500 cells per well in 6-well plates. When visible colonies appeared on the plate, 4% paraformaldehyde was added to fix the colonies for 20 min, followed by 0.5% crystal violet staining for 5 min. The number of colonies was counted under a microscope.

### Anchorage-independent cell growth assay

The soft agar assay was performed as described previously [16]. CRC cells were treated with or without irradiation (4 Gy), and then counted and suspended (8 × 10^3^ cells/well) in 1 ml 0.3% agar with 10% FBS Eagle’s medium. The cell suspension was seeded into a 6-well plate containing a 0.6% agar base and cultured at 37˚C in a 5% CO_2_ incubator for 2 weeks. The number of colonies was counted under a microscope.

### Clinical tissue sample collections

The present study was approved by the Research Ethics Committee of The Third Xiangya Hospital, Central South University (approval no. 2020-S518). All surgical specimens were collected in accordance with an Institutional Review Board-approved protocol. All subjects provided written informed consent for participation in the study. The patients included 80 cases of primary adenocarcinomas and matched non-tumor tissues, and 24 samples of relapse after radiotherapy.

### Immunohistochemical (IHC) staining

The IHC staining was performed as described previously [17]. Tissue sections were dewaxed and hydrated in gradient alcohols, and then immersed in boiling sodium citrate buffer (10 mM, pH 6.0) for 10 min for antigen repair, followed by treatment with 3% H_2_O_2_ for 10 min to block endogenous peroxidase activity. Next, blocking buffer containing 10% goat serum albumin was added to the slides and incubated for 1 h at room temperature. The tissues were then incubated with primary antibodies overnight at 4˚C, followed by hybridization with a secondary antibody for 30 min at room temperature. Hematoxylin was used for counterstaining. Slides were imaged with a light microscope and analyzed by using Image-Pro-PLUS software (version 6.2; Media Cybernetics, Inc.). The stained tissues were quantified based on a score that depended on the number of positive cells and the intensity of the staining. The percentage of positive cells was divided into four categories: 0, no positive cells; 1, ≤10% positive cells; 2, 10–50% positive cells; 3, >50% positive cells. The intensity was graded as 0, no staining; 1, weak staining; 2, moderate staining; 3, intense staining.

### Reverse transcription-quantitative PCR (RT-qPCR)

The primers for Bcl-xL were as follows: Forward sequence 5’-GCCACTTACCTGAATGACCACC-3’ and reverse sequence 5’-AACCAGCGGTTGAAGCGTTCCT-3’. For qPCR analysis, an ABI 7900HT systems (Applied Biosystems; This is a brand of Thermo Fisher Scientific, Inc.) was used with the following thermocycling conditions: phase 1: activation. 50 ˚C for 2 min; phase 2: presoak: 95 ˚C for 10 min; phase 3: denaturation: 95 ˚C for 15 s, and annealing: 60 ˚C for 1 min; phase 4: melting curve. 95 ˚C for 15 s, followed by 60 ˚C for 15 sec and 95 ˚C for 15 s.

### Protein preparation and western blotting

Whole-cell lysis from treated cells were extracted with RIPA buffer containing 10 mM Tris-Cl, pH 8.0, 1 mM EDTA, 0.5 mM EGTA, 1% Triton X-100, 0.1% sodium deoxycholate, 0.1% sodium dodecyl sulfate, and 140 mM NaCl. Lysis products were ultrasonicated and then centrifuged at 12,000 × *g* for 15 min at 4 ˚C. Protein concentrations were measured using BCA Assay Reagent (#23228, Thermo Fisher Science, Inc.). The western blotting was performed as described previously [15]. Protein samples (20 μg) were subjected to SDS-PAGE electrophoresis and IB analysis. Briefly, the proteins were transferred to a PVDF membrane and then incubated with 5% skimmed milk. Primary antibodies were incubated overnight at 4 ˚C, and secondary antibody was incubated for 30 min at room temperature. The immunoblot bands were observed with ECL reagents (#34579, Thermo Fisher Scientific, Inc.).

### Immunofluorescence (IF)

CRC cells were treated with IR (2 Gy) or not, fixed in ice-cold 4% paraformaldehyde and permeabilized in 0.5% Triton X-100 for 30 min. Next, the cells were blocked the cells with 10% goat serum albumin in PBS for 1 h at room temperature and then hybridized with a primary antibody against γ-H2AX overnight in a humidified chamber at 4 ˚C. The secondary antibody was then added for 40 min at room temperature. DAPI was used for nuclear staining. Visualization and capture of images was performed with a confocal fluorescence microscope (Nikon C1si; Nikon Corporation).

### Luciferase activity

CRC cells were transiently transfected with the pGL3-Basic vector or the pGL3-AP1 (#40342) construct for 48 h, followed by IR treatment for 1 h. Next, dual-Luciferase reporter assays were conducted as previously described [18]. All experiments were performed in triplicate with three independent experiments.

### Ubiquitination assay

Ubiquitination assay was performed as described previously [19]. Cells were lysed using RIPA buffer (20 mM NAP, 150 mM NaCl, pH 7.4, 0.5% sodium deoxycholate, 1% Triton and 1% SDS; PH 7.4) with 10 mM N-ethylmaleimide and protease inhibitors. Lysates were sonicated for 30 s, boiled at 95 ˚C for 5 min, diluted with RIPA buffer containing 0.1% SDS and centrifuged at 16,000 × *g* for 15 minutes at 4 ˚C. The supernatant was obtained and co-incubated with a primary antibody containing protein A-sepharose beads at 4 ˚C overnight. Samples were separated by SDS-PAGE and analyzed by western blotting.

### Tumor xenograft experiment

All mice were maintained and subjected to treatments according to the guidelines established by the Institutional Animal Care and Use Committee (No. 202009655) of Central South University, Changsha, China. Briefly, 6-week-old thymus-free female BALB/c-nude mice (strain no. D000521) were purchased from GemPharmatech Co. Ltd., Nanjing, China. Cells (1×10^6^) were suspended in 100 μl RPMI-1640 medium and injected into the right flank of nude mice (*n* = 5) to generate xenograft mouse models. When the tumor volume reached ~100 mm^3^, mice were treated with/without local IR (2 Gy/twice weekly) using X-RAD 320 (Precision X-ray, Inc.), which irradiates X-rays. For Bcl-xL inhibitor A-1155463 treatment, the tumor-bearing mice were randomly divided into four groups (*n* = 5): 1, vehicle control (0.5% dimethyl sulfoxide, 100 mL/every 2 days, i.p.); 2, local IR (2 Gy/ twice per week); 3, A-1155463 (5 mg/kg/ every 2 days, i.p.); 4, A-1155463 (5 mg/kg/ every 2 days, i.p.) + local IR (2 Gy/ twice per week). Tumor volumes were recorded and calculated using the following formula: Volume = (length x width x width/2). Finally, the mice were euthanized, and the tumor tissues were collected for IHC staining.

### Statistical analysis

SPSS (version16.0 for Windows, SPSS Inc, Chicago, IL, USA) and GraphPad Prism 5 (version 5.0; GraphPad Software; Dotmatics) were used for data analysis. Student’s t-test or ANOVA was used to assess the differences between the means. Pearson rank correlation was used for correlation analysis. The difference in expression between the adjacent and tumor tissues was evaluated by Wilcoxon signed-rank test. All quantitative data are expressed as the mean ± SD of three independent experiments. For all statistical analyses, *p* < 0.05 was considered to indicate a statistically significant difference.

## Results

### TRAF4 knockout increases the sensitivity of CRC cells to irradiation

**Fig. 1:**
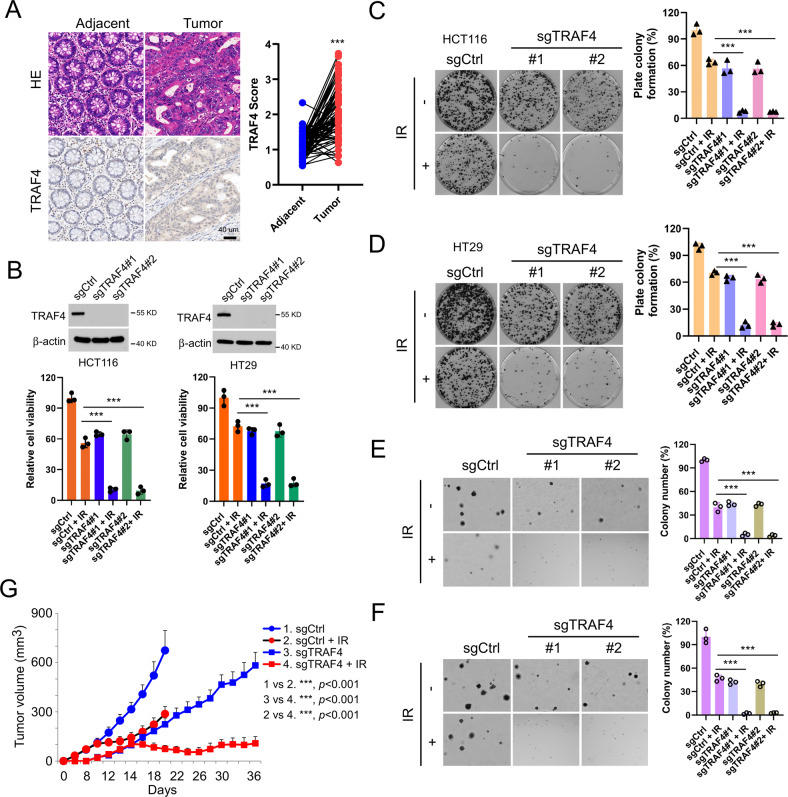
TRAF4 knockout increases the sensitivity of CRC cells to irradiation. **A** IHC staining was performed to determine TRAF4 expression in 80 cases of primary CRC tumors with matched adjacent tissues. Scale bar, 40 μm. ***p < 0.001. **B** Immunoblotting (IB) for TRAF4 expression in sgTRAF4-HCT116 and -HT29 cells. MTS assay was used to determine the viability of sgTRAF4-HCT116 and -HT29 cells with or without IR (4 Gy) treatment. **C** and **D**. Plate colony formation of sgTRAF4-HCT116 (**C**) and -HT29 (**D**) cells with or without IR (4 Gy) treatment. ***p < 0.001. E and F. Soft agar assay was conducted to assess the anchorage-independent cell proliferation of sgTRAF4-HCT116 (**E**) and -HT29 (**F**) cells with or without IR (4 Gy) treatment. ***p < 0.001. **G** Average tumor volume of HCT116-sgCtrl and HCT116-sgTRAF4 xenograft tumors with or without IR (2 Gy/ twice per week) treatment; *n* = 5 mice per group. ***p < 0.001. TRAF4 TNF receptor-associated factor 4, CRC colorectal cancer, IR ionizing radiation.

To determine the function of TRAF4 on radiotherapy, the expression of TRAF4 was first analyzed in CRC tissues using IHC staining. As shown in Fig. 1A, compared with the findings in paired adjacent tissues, TRAF4 protein was overexpressed in CRC tissues. Next, stable TRAF4-knockout HCT116 and HT29 cells were generated (Fig. 1B). The data showed that TRAF4 depletion significantly suppressed the proliferation of CRC cells in the presence of irradiation (2 Gy), as demonstrated by decreased cell viability (Fig. 1B) and plate colony formation ability (Fig. 1C and D), and impaired anchorage-independent cell proliferation potential (Fig. 1E and F). Xenografts studies also revealed that depletion of TRAF4 led to a remarkable reduction in HCT116 cell proliferation with irradiation treatment in vivo compared with that of xenograft tumors retaining TRAF4 with/without irradiation treatment or TRAF4 knockout-xenograft tumors that did not receive the irradiation (Fig. 1G and S1). These findings indicated that TRAF4 was required for maintaining the tumorigenic properties of CRC cells, and blocking TRAF4 expression increased the sensitivity of CRC cells to irradiation.

### TRAF4 knockout enhances IR-induced intrinsic apoptosis

**Fig. 2:**
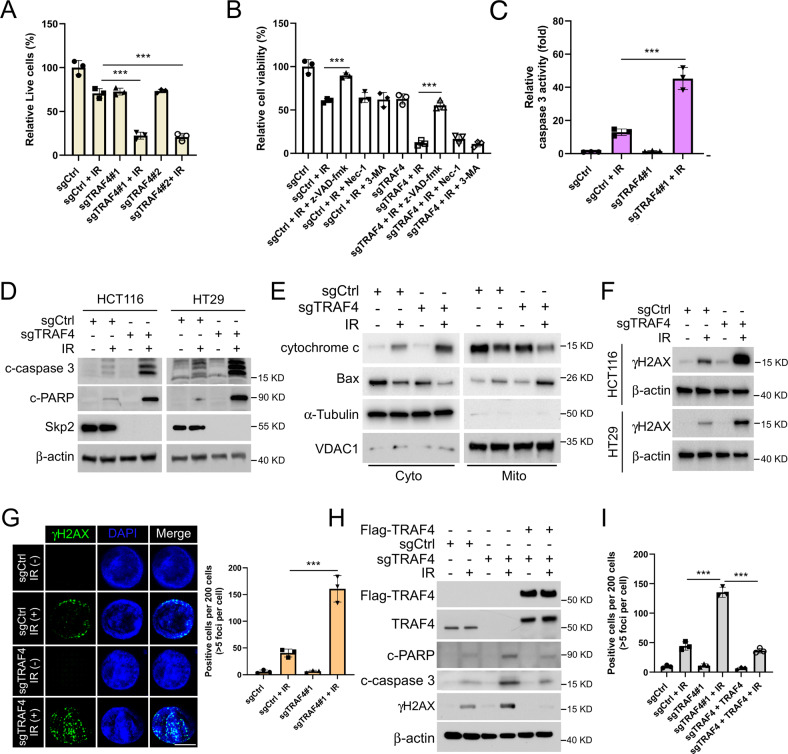
TRAF4 knockout enhances IR-induced intrinsic apoptosis. **A** TRAF4-null HT29 cells were treated with or without IR (4 Gy) and cultured for 72 h. The population of live cells was determined by trypan blue exclusion assay. ***p < 0.001. **B** TRAF4-null HT29 cells were pretreated with the apoptosis inhibitor z-VAD-fmk, necroptosis inhibitor necrostatin-1, and autophagy inhibitor 3-MA for 4 h, followed by IR (4 Gy) treatment and cultured for 72 h. Cell viability was detected by MTS assay. ***p < 0.001. **C**–**E** TRAF4-null HT29 cells were treated with or without IR (4 Gy) and cultured for 72 h. The Caspase 3 Assay Kit was used to analyze caspase 3 activity (**C**), and the protein level of cleaved-caspase 3 and cleaved-PARP was examined by IB analysis (**D**), the subcellular fractions were isolated to examine the expression of Bax and cytochrome c (**E**). ***p < 0.001. **F** and **G** Immunoblotting (**F**) and Immunofluorescent (**G**) for γ-H2AX expression in sgTRAF4-HCT116 and -HT29 cells with or without IR (4 Gy) treatment for 72 h. Scale bar, 5 μm. H and I. TRAF4-null HCT116 cells were transfected with Flag-TRAF4 for 24 h, then treated with IR (4 Gy) and cultured for 72 h. IB was used to examine the protein expression (H); IF quantitative analysis was performed to determine γ-H2AX expression (**I**). ***p < 0.001. TRAF4 TNF receptor-associated factor 4, IR ionizing radiation, IB immunoblotting, γ-H2AX γ - H2A histone family member X.

Trypan blue exclusion assay showed that IR treatment increased the population of dead cells in TRAF4-knockout CRC cells (Fig. 2A and S2A). We next determined which cell death pathway was associated with IR-induced cell death in TRAF4-knockout CRC cells. The three most common cell death inhibitors, such as apoptosis inhibitor z-VAD-fmk, necroptosis inhibitor necrostatin-1 and autophagy inhibitor 3-MA, were used to pretreat TRAF4-null CRC cells. As shown in Fig. 2B and S2B, the apoptosis inhibitor z-VAD-fmk restored cell viability significantly in the presence of irradiation, suggesting that activation of apoptosis was the main pathway of IR-induced CRC cell death. Furthermore, the activity of caspase 3 induced by irradiation was upregulated noticeably in TRAF4-knockout cells (Fig. 2C and S2C). By analyzing the protein expression levels of cleaved-caspase 3 and -PARP, IB data demonstrated that blocking TRAF4 increased cleaved-caspase 3 and -PARP expression following treatment with irradiation (Fig. 2D). The subcellular fractions assay revealed that release of cytochrome *c* from mitochondria and Bax translocation to mitochondria were increased in the presence of irradiation, and both effects were further enhanced upon TRAF4 depletion (Fig. 2E). Of note, the protein expression levels of the DNA damage marker γ-H2AX protein was substantially upregulated in TRAF4-knockout HCT116 and HT29 cells treated with irradiation (Fig. 2F). Similar results were observed in the immunofluorescence assay with γ-H2AX staining (Fig. 2G). Moreover, the reintroduction of TRAF4 in TRAF4-null HCT116 cells compromised IR-induced expression of cleaved-caspase 3, cleaved-PARP and γ-H2AX (Fig. 2H and I). These data suggested that depletion of TRAF4 enhanced the IR-activated intrinsic apoptotic pathways and DNA damage in CRC cells.

### Bcl-xL is required for IR-induced apoptosis in TRAF4-knockout CRC cells

**Fig. 3:**
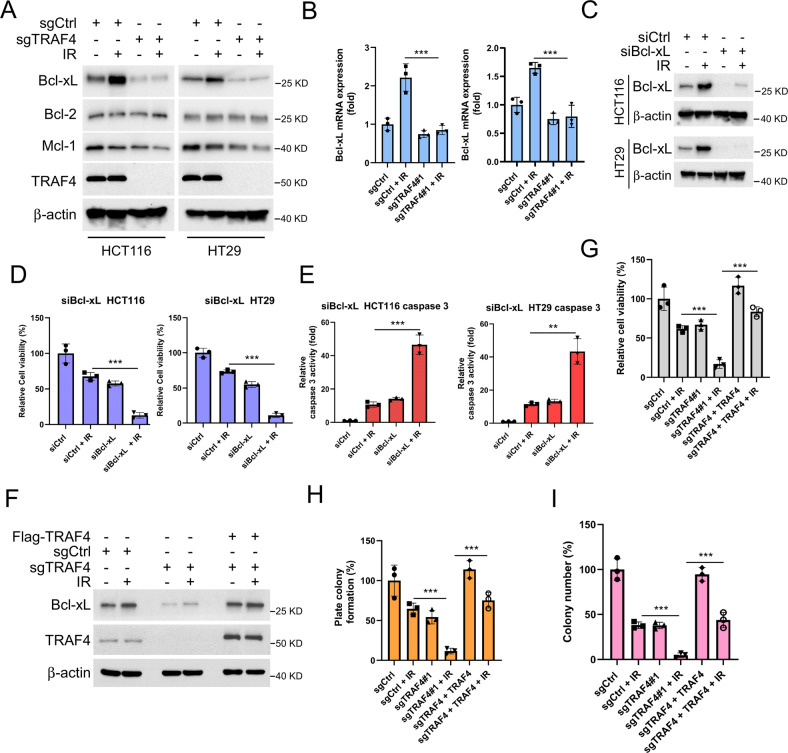
TRAF4 Knockout reduces IR-induced Bcl-xL expression. **A** and **B**. TRAF4-null HCT116 and -HT29 cells were treated with or without IR (4 Gy) and cultured for 4 h, Whole-cell extract (WCE) was subjected to IB analysis (**A**); the mRNA level of Bcl-xL was examined by RT-qPCR (B). ***p < 0.001. C-E. SiBcl-xL was transfected into HCT116 and HT29 cells for 24 h, followed by IR (4 Gy) treatment and cultured for 4 h. IB was used to examine the proteins expression (**C**); MTS assay was performed to detect the cell viability (**D**); Caspase 3 Assay Kit was used to determine caspase 3 activity (E). ***p < 0.001. **p < 0.01.  F-I. Flag-TRAF4 was transfected into sgTRAF4-HCT116 cells for 24 h, followed by IR (4 Gy) treatment, and cultured for 72 h. IB was used to examine the expression of the protein (F); MTS assay was performed to detect the cell viability (**G**); Plate colony formation assay was performed to analyze the cell proliferation capacity (**H**); Soft agar assay was conducted to assess the anchorage-independent cell growth ability (**I**). ***p < 0.001. TRAF4 TNF receptor-associated factor 4, IR ionizing radiation IB immunoblotting, WCE whole-cell extract.

To determine the underlying mechanism of IR-induced apoptosis, the antiapoptotic Bcl-2 family members in TRAF4-depleted HCT116 and HT29 cells were examined. The results showed that Bcl-xL expression was upregulated after irradiation treatment, which was consistent with several recent lines of evidence [20]. However, depletion of TRAF4 attenuated the upregulation of Bcl-xL expression (Fig. 3A). The mRNA level of Bcl-xL was in line with that of its protein expression according to the results of RT-qPCR assay (Fig. 3B), suggesting that TRAF4 may regulate the expression of Bcl-xL in response to irradiation. Bcl-xL-knockdown cells were then constructed by transfecting siRNA into HCT116 and HT29 cells (Fig. 3C). The results showed that knockdown of Bcl-xL and exposure to irradiation significantly reduced cell viability (Fig. 3D), and increased caspase 3 activity (Fig. 3E) in CRC cells. Ectopic overexpression of TRAF4 in TRAF4-knockout CRC cells restored the protein levels of Bcl-xL (Fig. 3F), as well as cell viability (Fig. 3G and S3A) and colony formation ability on plate or in soft agar (Fig. 3H and I, and S3B and C) even in the presence of irradiation. Furthermore, the reintroduction of Bcl-xL into TRAF4-null HT29 cells compromised the IR-induced upregulation of cleaved-caspase 3 and -PARP (Fig. S3D), and dramatically increased the cell viability (Fig. S3E). These results indicated that Bcl-xL was essential for IR-induced intrinsic apoptosis in TRAF4-deficient CRC cells.

### TRAF4 is required for IR-induced c-Jun expression

**Fig. 4:**
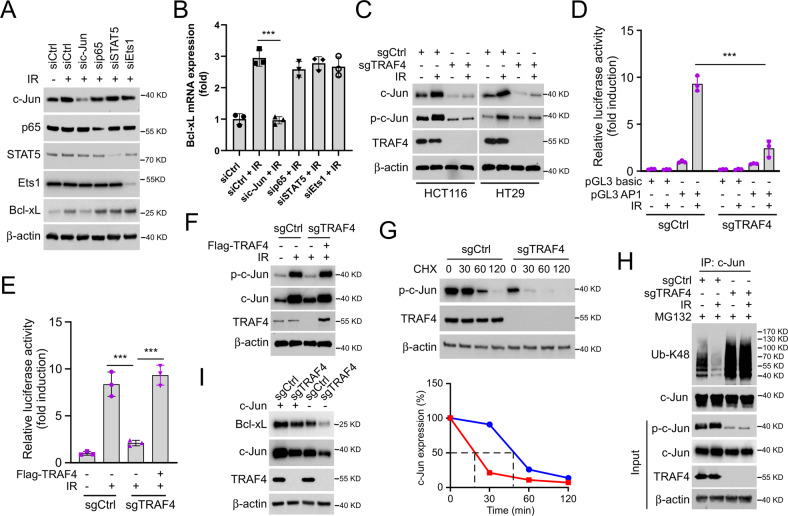
c-Jun is required for IR-induced Bcl-xL expression. **A** and **B** HT29 cells were transfected with sic-Jun, sip65, siSTAT5 and siEst1 for 24 h, followed by IR (4 Gy) treatment and cultured for 4 h. IB was used to examine the protein expression (**A**); the mRNA levels of Bcl-xL was examined by RT-qPCR (B). ***p < 0.001. **C** TRAF4-null HCT116 and -HT29 cells were treated with or without IR (4 Gy) and cultured for 30 min. IB was used to examine the protein expression. **D** TRAF4-null HT29 cells were transfected with pGL3 basic or pGL3 AP1 for 48 h and treated with or without IR (4 Gy). Reporter activity was examined 30 min later. ***p < 0.001. **E** and **F** TRAF4-null HT29 cells were transfected with Flag-TRAF4 for 48 h, followed by IR (4 Gy) treatment. Reporter activity (**E**) and protein expression (**F**) were examined 30 min later. ***p < 0.001. G. TRAF4-null HT29 cells were treated with cycloheximide for various time points, and IB was used to examine the protein expression. **H** TRAF4-null HT29 cells were pretreated with MG132 for 6 h, followed by IR (4 Gy) treatment for 30 min, and WCE was subjected to ubiquitination analysis. **I** TRAF4-null HT29 cells were overexpressed with c-Jun for 48 h, and WCE was subjected to IB analysis. TRAF4 TNF receptor-associated factor 4, IR ionizing radiation, IB immunoblotting, siRNA small interfering RNA, AP-1 activator protein-1, WCE whole-cell extract.

As we had observed, TRAF4 knockout reduced the expression of Bcl-xL by affecting its transcriptional process. Multiple transcription factors have been demonstrated to regulate Bcl-xL expression, including STATs, c-Jun, Rel/NF-kB p65 and Ets [21]. To investigate which of these transcription factors were associated with IR-induced upregulation of Bcl-xL, their expression was silenced by transfecting siRNA, and it was found that knockdown of c-Jun, but not of p65, STAT5 or Ets1, impaired the protein and mRNA levels of Bcl-xL in HT29 cells with irradiation treatment (Fig. 4A and B). Next, the levels of phosphorylated and total c-Jun were determined by IB analysis, which revealed that TRAF4 knockout strongly decreased irradiation-induced c-Jun and p-c-Jun expression (Fig. 4C). In agreement with this result, the luciferase activity mediated by activator protein-1 (AP-1), whose activation required the dimerization of the c-Jun, c-Fos and Fra families, was significantly increased with irradiation treatment, whereas it was compromised following TRAF4 depletion (Fig. 4D and S4). Furthermore, the functional activity of luciferase (Fig. 4E), the expression of the phosphorylated and total forms of c-Jun (Fig. 4F) was markedly increased after ectopic overexpression of TRAF4 in TRAF4-null HT29 cells. Moreover, the depletion of TRAF4 shortened the half-life of c-Jun prominently (Fig. 4G). Notably, the ubiquitination level of c-Jun was determined by ubiquitination assay, and was strongly increased in TRAF4-knockout cells (Fig. 4H), suggesting that TRAF4 was also associated with the post-translational modification of c-Jun. Expectedly, as shown in Fig. 4I, depletion of TRAF4 reduced the protein level of c-Jun and Bcl-xL, and overexpression of c-Jun restored Bcl-xL expression in TRAF4-null HT29 cells. These findings supported that TRAF4-mediated c-Jun upregulation was required for IR-induced Bcl-xL expression in CRC cells.

### TRAF4 promotes JNK1/2 ubiquitination, which is essential for IR-induced JNK/c-Jun signaling activation

**Fig. 5:**
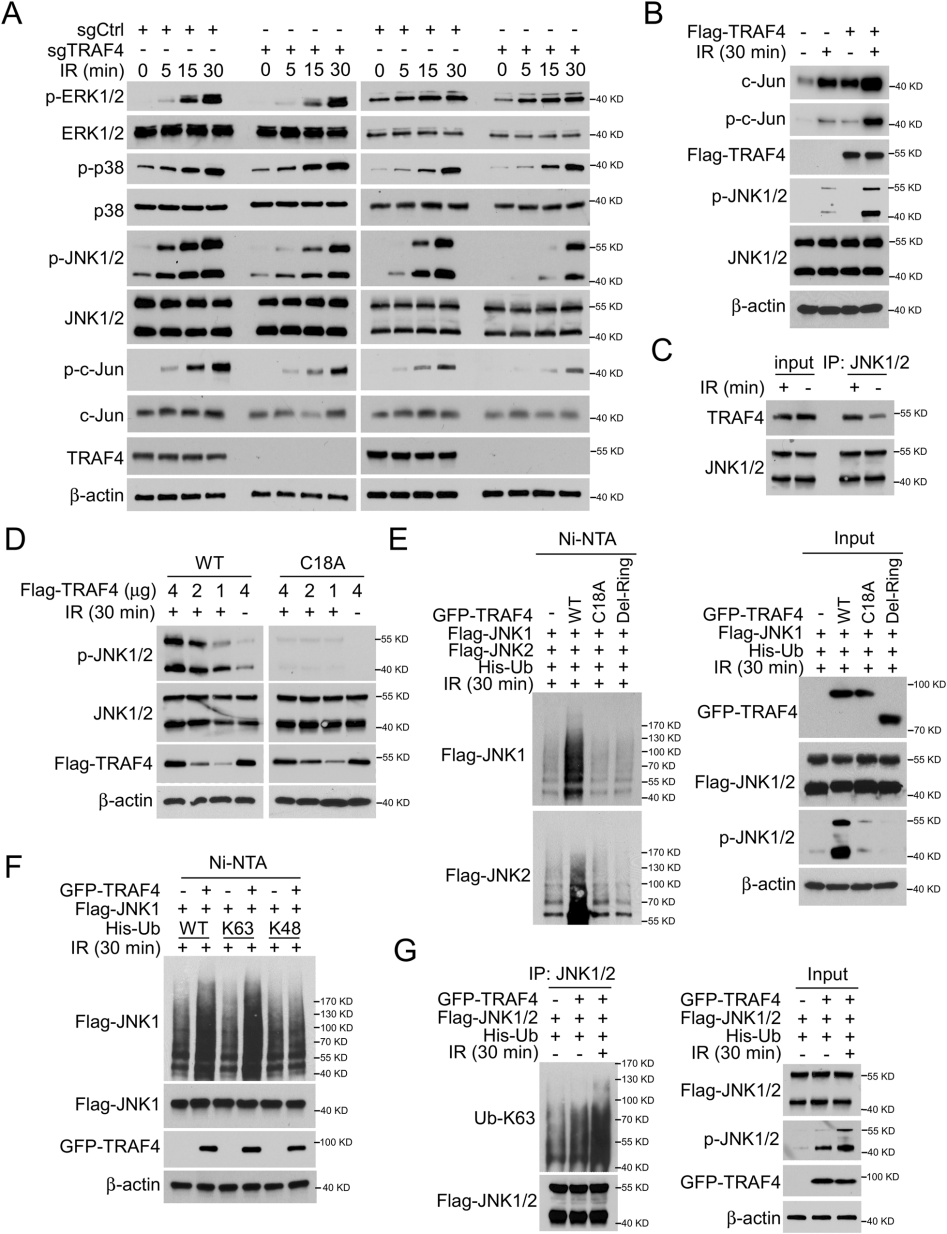
TRAF4 activates JNK/c-Jun signaling. **A** TRAF4-null HCT116 and HT29 cells were treated with or without IR (4 Gy) for various time points, and WCE was subjected to IB analysis. **B** Flag-TRAF4 was transfected into FHC non-tumor cells for 48 h, followed by IR (4 Gy) treatment for 30 min. WCE was subjected to IB analysis. **C** HT29 cells were treated with or without IR (4 Gy) for 30 min, WCE was subjected to co-IP assay. **D** TRAF4-knockout HT29 cells were transfected with Flag-TRAF4 WT or Flag-TRAF4 C18A mutant for 48 h, followed by IR (4 Gy) treatment for 30 min. WCE was subjected to IB analysis. E. Flag-JNK1/2 and His-Ub were co-transfected with GFP-TRAF4 WT, GFP-TRAF4 C18A mutant or Del-Ring in 293 T cells for 48 h, then treated with IR (4 Gy) for 30 min. Ni-NTA pull-down assay was performed to detect JNK1/2 ubiquitination. F. 293 T cells were co-transfected with GFP-TRAF4, Flag-JNK1 and His-Ub WT or mutants for 48 h, then treated with IR (4 Gy) for 30 min. Ni-NTA pull-down assay was performed to detect JNK1 ubiquitination. G. HT29 cells were co-transfected with GFP-TRAF4, Flag-JNK1/2, and His-Ub for 48 h, then treated with or without IR (4 Gy) for 30 min. WCE was subjected to IP and ubiquitination analyses. TRAF4 TNF receptor-associated factor 4, IR ionizing radiation, IB immunoblotting, WT wild-type, NTA nitriloacetic acid, IP immunoprecipitation, WCE whole-cell extract, Ub ubquitination.

Next, the present study determined the mechanism by which TRAF4 regulates IR-induced c-Jun expression. As shown in Fig. 5A, the phosphorylation of JNK1/2 was reduced substantially in TRAF4-null HCT116 and HT29 cells. However, the activation of p38 and ERK1/2 was unchanged in TRAF4 wild-type (WT)/null CRC cells. In addition, overexpression of TRAF4 increased IR-induced upregulation of p-c-Jun and p-JNK1/2 expression (Fig. 5B). The interaction between TRAF4 and JNK1/2 was demonstrated to be enhanced in the presence of irradiation (Fig. 5C). TRAF4 WT, but not the mutation responsible for its catalytic inactivation (C18A), activated JNK1/2 in the present of irradiation (Fig. 5D). Ubiquitination analysis showed that WT TRAF4 increased the ubiquitination and phosphorylation of JNK1/2 after IR treatment. However, these post-translational modifications were markedly abrogated in TRAF4 C18A or RING domain-deleted (Del-Ring) transfected cells (Fig. 5E), indicating that the E3 ligase activity of TRAF4 was required for IR-induced activation of JNK1/2. Moreover, co-transfection of TRAF4 and ubiquitin (WT) or K48 (lysine 48 only)/K63 (lysine 63 only) mutants further revealed that the TRAF4-induced JNK1 ubiquitination was K63-linked (Fig. 5F). Consistently, the ubiquitin assay showed that the K63-linked ubiquitination of JNK1/2 was upregulated robustly with TRAF4 overexpression in response to irradiation (Fig. 5G). The present data revealed that TRAF4-mediated K63-linked ubiquitination was essential for IR-induced JNK1/2 activation.

### TRAF4 knockout sensitizes radiotherapy in vivo

**Fig. 6:**
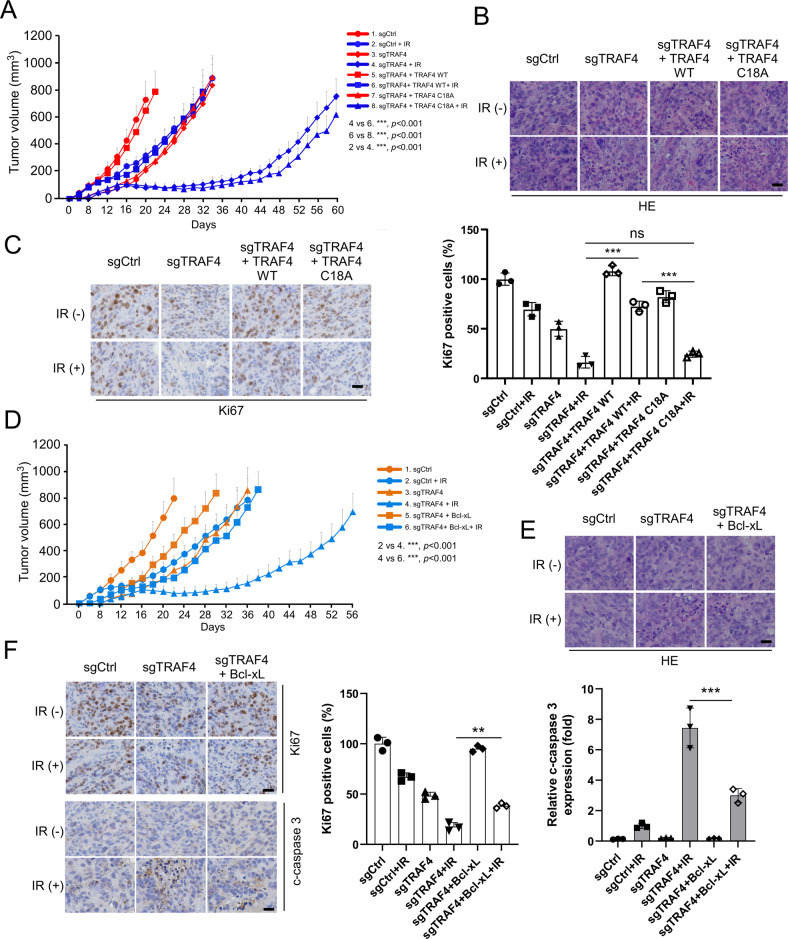
TRAF4 knockout combined with irradiation inhibits tumor growth in vivo. **A**-**C**. TRAF4 reintroduction into TRAF4-null HT29 cells rescues tumorigenesis under IR treatment (2 Gy/twice per week). TRAF4 WT or TRAF4 C18A mutant was reintroduced into TRAF4 null-HT29 cells and subjected to in vivo tumorigenesis. (*n* = 5 mice per group). Tumor growth curves (**A**) were recorded, tumor tissues were subjected to H&E (**B**) and IHC staining (**C**). For C, left, representative image; right, qualification. Scale bar, 25 μm. ***p < 0.001. **D**–**F**. Introduction of Bcl-xL into TRAF4-null HT29 cells rescues tumorigenesis under IR treatment (2 Gy/twice per week). Bcl-xL was introduced into TRAF4 knockout-HT29 cells and subjected to in vivo tumorigenesis. (*n* = 5 mice per group). Tumor growth curves (**D**) were recorded, tumor tissues were subjected to H&E (**E**) and IHC staining (**F**). For **F**, left, representative image; middle and right, qualification for Ki67 (middle) and cleaved-caspase 3 (right). Scale bar, 25 μm.***p < 0.001. **p < 0.01. TNF receptor-associated factor 4, IR ionizing radiation, H&E hematoxylin and eosin, IHC immunohistochemistry.

To validate the effect of TRAF4 on the radiotherapy of CRC cells in vivo, xenograft tumor models were generated using HT29 cells. Compared with that of tumors derived from TRAF4-knockout cells that were not exposed to irradiation or with tumors with TRAF4 WT restored and treated with irradiation, tumor growth was significantly inhibited in xenograft tumors from TRAF4-knockout cells subjected to irradiation. Importantly, in TRAF4 knockout-xenograft tumors, the reintroduction of the TRAF4 C18A mutant did not compromise the antitumor effect of irradiation (Fig. 6A), indicating that the E3 ligase activity of TRAF4 was required for radioresistance in CRC cells. Hematoxylin and eosin (HE) and IHC staining showed that the population of Ki67-positive cells was significantly reduced in IR-treated TRAF4-null tumors. Overexpression of TRAF4, but not of TRAF4 C18A, restored the expression of Ki67 in tumor cells after radiotherapy (Fig. 6B and C). Next, the impact of Bcl-xL on the radiotherapeutic function was investigated. In TRAF4-knockout xenograft tumors, the introduction of Bcl-xL markedly attenuated the inhibitory effect of irradiation and rescued tumorigenesis in TRAF4-null CRC cells, even with irradiation treatment (Fig. 6D). Moreover, the reintroduction of Bcl-xL increased the population of Ki67 positive cells, and reduced IR-induced apoptosis in TRAF4-null tumors, since the cleaved-caspase 3 was reduced significantly (Fig. 6E and F). These results suggested that depletion of TRAF4 increased the sensitivity of CRC cells to radiotherapy, and the mechanism may be associated with the reduction of Bcl-xL in TRAF4-null cells.

### TRAF4 is upregulated and positively correlates with Bcl-xL in samples of patient with CRC

**Fig. 7:**
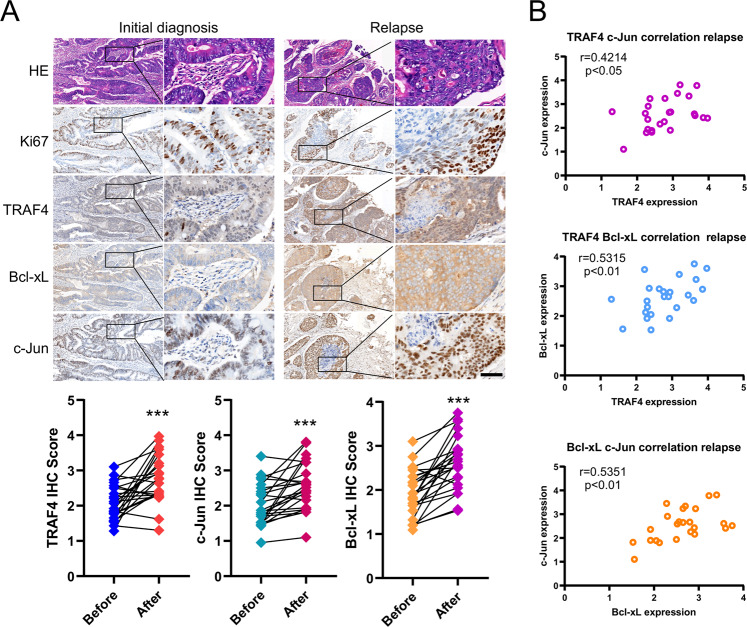
TRAF4 is upregulated and positively correlates with Bcl-xL in samples of patients with colorectal cancer. **A** Top, IHC staining was performed to determine Ki67, TRAF4, Bcl-xL and c-Jun in 24 cases paired primary and relapsed tumor tissues. Bottom, Image-Pro-PLUS (v.6) and Image J (NIH) computer software were used to quantify staining intensity. ***p < 0.001. Scale bar, 50 μm. **B** Scatterplot shows the positive correlation between c-Jun and TRAF4 (top), Bcl-xL and TRAF4 (middle), c-Jun and Bcl-xL (bottom) expression in the relapsed tumor.

To verify the clinical relevance of the present findings, we firstly analyzed the expression and prognostic value of TRAF4 and Bcl-xL in CRC using the bioinformatics web tools of the Gene Expression Profiling Interactive Analysis (GEPIA) and Kaplan-Meier plotter. The mRNA levels of TRAF4 and Bcl-xL were upregulated in colon adenocarcinoma (COAD) and rectum adenocarcinoma (READ) (Fig. S5A and B), and exhibited a positive correlation in CRC (Fig. S5C). Moreover, Kaplan-Meier survival analysis showed that CRC patients with higher TRAF4 and Bcl-xL expression had a worse prognosis (Fig. S5D and E). Subsequently, the expression of TRAF4, c-Jun and Bcl-xL was examined in primary CRC tumor tissues. Compared with those in paired adjacent non-tumor tissues, the TRAF4, c-Jun and Bcl-xL protein levels were significantly upregulated (Fig. S5F). Furthermore, IHC staining showed that the expression of TRAF4 was positively correlated with c-Jun and Bcl-xL. There was also a positive correlation between c-Jun and Bcl-xL expression (Fig. S5G). Notably, we next evaluated the TRAF4, c-Jun and Bcl-xL expression in 24 relapsed CRC specimens by IHC staining. Moreover, the IHC staining data revealed that these three proteins were significantly higher in relapsed tumor tissues than that in the initially diagnosed samples (Fig. 7A), and the positive correlation among TRAF4, c-Jun and Bcl-xL proteins was also observed in the relapsed tumor tissues (Fig. 7B). These data supported that TRAF4 and its downstream proteins c-Jun and Bcl-xL were overexpressed in primary CRC tissues, and further highly expressed in relapsed tumor tissues, which provided a potential mechanism for tumorigenesis and radioresistance in CRC.

### Inhibiting the TRAF4/ Bcl-xL axis overcomes radioresistance in CRC cells

**Fig. 8:**
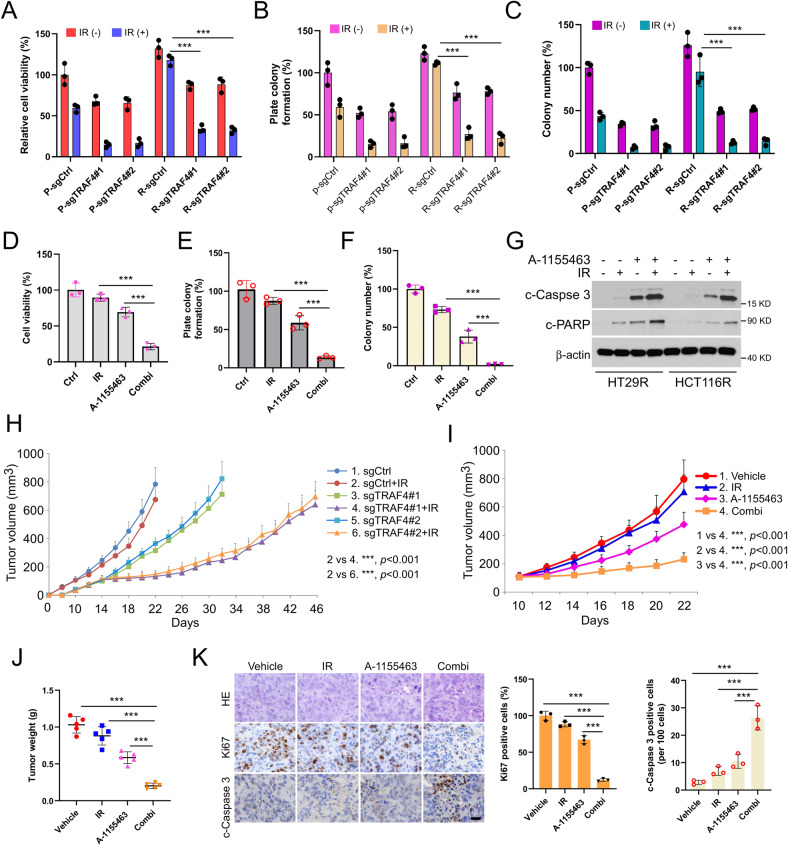
Inhibiting the TRAF4/ Bcl-xL axis overcomes radioresistance in colorectal cancer cells. **A–C** Parental and radioresistant HT29 cells expressing sg-Ctrl or sg-TRAF4 were treated with or without IR (4 Gy). Cell viability and colony formation were determined by MTS (**A**), plate colony formation (**B**), and soft agar (**C**) assays. P, parental cells; R, resistant cells. ***p < 0.001. **D**–**G** HT29R cells were treated with A-1155463 inhibitor (2 µM), IR (4 Gy), or a combination of both for 72 h. MTS assay was used to determine the cell viability (**D**), plate colony formation assay to analyze the cell proliferation (**E**), and soft agar assay to assess the anchorage-independent cell proliferation (**F**). Immunoblotting to detect the expression of cleaved-caspase 3 and cleaved-PARP (**G**). ***p < 0.001. H. Average tumor volume of HT29R-sgCtrl and -sgTRAF4 xenograft tumors treated with or without IR (2 Gy/twice per week); *n* = 5 mice per group. I-K. Xenograft tumors from HT29R cells were treated with the vehicle control, A-1155463 (5 mg/kg/every 2 days), IR (2 Gy/twice per week), or a combination of both; *n* = 5 mice per group. Average tumor volume (**I**) and tumor weight (**J**) were recorded, and tumor sections were subjected to IHC staining of Ki67 and cleaved-caspase 3 (**K**). For K, left, representative image; right, qualification. ***p < 0.001. Scale bar, 25 μm. TRAF4 TNF receptor-associated factor 4, IR ionizing radiation, Ctrl control, P parental cells, R resistant cells.

We next generated radioresistant CRC cell lines to determine the role of TRAF4/Bcl-xL signaling in the radioresistance of CRC cells (Fig. S6A). In HT29R1 and HCT116R7 radiotherapy-resistant colonies, the high level of TRAF4 was accompanied by an upregulated Bcl-xL expression compared with the findings in parental sensitive cells, we therefore focused on these two colonies, which were termed HT29R and HCT116R, respectively, for further experiments. Stable TRAF4-knockout cell lines were generated using HT29R and HCT116R cells (Fig. S6B). The cell viability of parental and radioresistant CRC cells treated with irradiation was evaluated by MTS assay. The results showed that TRAF4 depletion significantly recovered the antitumor effect of irradiation in radioresistant CRC cells (Fig. 8A and S6C). As expected, colony formation on the plate or in soft agar was also impaired following TRAF4 knockout in radioresistant CRC cell (Fig. 8B and C, and S6D and E). Moreover, the specific small molecule inhibitor of Bcl-xL, A-1155463, was used in subsequent experiments in vitro and in vivo. The results indicated that the combination of A-1155463 and irradiation significantly suppressed cell viability (Fig. 8D and S6F) and colony formation abilities (Fig. 8E and F, and S6G and H) in radioresistant CRC cells compared with those observed in cells treated with A-1155463 or irradiation alone. Intrinsic apoptosis was activated with A-1155463 treatment and further enhanced in combination with irradiation, since the IB data showed that the expression of cleaved-Caspase 3 and cleaved-PARP was increased obviously (Fig. 8G). We then generated the xenograft tumors using HT29R cells and found that tumors from TRAF4-knockout cells combined with irradiation exhibited the lowest tumor growth rate (Fig. 8H). Additionally, xenograft tumors derived from HT29R cells were administrated irradiation, A-1155463, or combination treatment. It was observed that the combination of A-1155463 and irradiation delayed the growth of tumors substantially (Fig. 8I and J). Consistently, the population of Ki67-positive cells was significantly decreased and the expression of cleaved-caspase 3 was markedly increased, as indicated by IHC staining (Fig. 8K). Altogether, these results suggested that dysregulation of Bcl-xL-mediated apoptosis played a critical role in the radioresistance of CRC cells, and targeting the TRAF4/Bcl-xL axis may be a promising therapeutic strategy to overcome the radioresistance of CRC cells.

## Discussion

TRAF4, a vital member of the TRAF family, was initially identified as a differential gene highly expressed in breast cancer [22]. Overexpression or amplification of TRAF4 has been observed in lung cancer [11], osteosarcoma [23], glioma [24] and other tumors. In breast cancer, TRAF4 inhibits apoptosis and promotes tumor cell proliferation by suppressing the ubiquitination of the spindle assembly-associated protein Eg5 [25]. In addition, TRAF4 activated the PI3K/AKT signaling pathway to promote the migration and invasion of hepatocellular carcinoma cells [26], and clinical evidence suggested that TRAF4 was closely associated with poor prognosis in intrahepatic cholangiocarcinoma [27]. Overexpression of TRAF4 led to trastuzumab resistance by activating HER2 signaling in HER2-positive breast cancer [28]. In glioblastoma (GBM), TRAF4 regulates caveolin 1 (CAV1) stability, and drives GBM cell stemness and temozolomide resistance by blocking ZNRF1-mediated ubiquitination and promoting USP7-mediated deubiquitination [29]. In addition, TRAF4 could reduce the growth inhibitory effect of etoposide by reducing the number of S-phase cells in breast cancer cells and inhibiting apoptosis [30], which may be the reason for the poor chemotherapy efficiency in patients with breast cancer with high TRAF4 expression. The present study found that TRAF4 is required for maintaining the tumorigenic properties of CRC. Knockout of TRAF4-sensitized radiotherapy and promoted IR-induced apoptosis by reducing Bcl-xL expression. Notably, it has been demonstrated that stereotactic radiation therapy (SRT) is an effective and minimally invasive treatment strategy for advanced CRC, especially in lung-limited oligo-metastatic (om) patients [31–33]. Based on our findings, the further study focuses on whether blocking TRAF4 combined with SRT treatment has a positive synergistic effect in promoting tumor regression and preventing tumor recurrence may be a clinically valuable research point that provides a potential management strategy for omCRC patients.

Bcl-xL is an antiapoptotic protein of the Bcl-2 family that binds proapoptotic proteins such as Bax and Bad through the BH4 structural domain to inhibit the activation of apoptotic signals [34]. Dysfunction of apoptosis signaling confers chemoresistance in cancer therapy. It is frequently observed that overexpression of antiapoptotic proteins counteracts the antitumor efficacy of therapeutic agents [35]. It was shown that Bcl-xL overexpression via retroviral transduction led to reduced sensitivity to adriamycin in high-risk B-cell acute lymphoblastic leukemia cell lines [36]. In metastatic bladder cancer, Bcl-xL protein was highly expressed in cisplatin-resistant UC cells (T24/R). The events of cisplatin-induced cytotoxicity and DNA damage were restored in T24/R cells with Bcl-xL knockdown by transfecting siRNA [37]. Acquired radioresistance remains an obstacle to overcome in current radiotherapy. Head and neck squamous cell carcinoma and synovial sarcoma cell lines with high Bcl-xL expression levels were more resistant to radiation therapy [20]. Stable expression of Bcl-xL in lung cancer cells has been shown to protect cancer cells from short- and long-term radiation effects. By contrast, high expression of Bcl-xL promotes the propagation of cancer cells with a radiation-resistant phenotype by enhancing alternative end-joining and homologous recombination repair during double-strand break treatment [38]. The current study identified Bcl-xL as a TRAF4 downstream gene regulated by TRAF4-mediated JNK/c-Jun signaling activation. Bcl-xL overexpression reversed the apoptotic phenotype in radiation-treated TRAF4-null CRC cells, and combination with Bcl-xL inhibitor overcame radioresistance. This result provides new insights to elucidate the cause of radioresistance in colon cancer.

JNKs are members of the MAPK signaling pathway, which is activated by extracellular stimuli such as stress and radiation, and subsequently phosphorylates and activates the early response transcription factor c-Jun, a component of the activator protein 1 (AP-1) complex [39]. It is mainly involved in various cellular processes such as cell proliferation, apoptosis, angiogenesis and inflammation [40]. A previous study has shown that JNK has opposite functions in regulating tumor development. In vestibular nerve sheath tumor cells [41], JNK activity protects against apoptosis by inhibiting the accumulation of superoxide in mitochondria. However, vitamin K2 induced mitochondria-associated apoptosis via the ROS-JNK/p38 MAPK signaling pathway in bladder cancer [42]. Therefore, a more profound elucidation of the JNK signaling pathway may help precision target cancer and generate more effective clinical outcomes. JNK also greatly influences radiotherapy tolerance and chemoresistance during cancer treatment. Recent study have demonstrated that inhibition of JNK and PARP1 can reverse miRNA-363-3p-associated doxorubicin resistance in diffuse large B-cell lymphoma [43]. Depleting STAMBPL1 and MKP-1 can enhance the sensitivity of breast cancer cells to cisplatin by increasing JNK phosphorylation and activation [44]. In addition, upregulation of Tiam1 expression could also promote radioresistance in laryngeal squamous cell carcinoma by activating the JNK/ATF-2 signaling [34]. The current study found that TRAF4 induced degradation-independent K63-linked ubiquitination on JNK1/2 to promote its kinase activity in CRC cells. Our study revealed for the first time that ubiquitination regulated JNK activation in mammalian cells, which was consistent with a previous study reporting that Tankyrase mediates K63-Linked ubiquitination activated JNK and confered stress tolerance in *Drosophila* [45].

Overall, this study provided a new underlying mechanism for TRAF4-mediated radioresistance, suggesting that the TRAF4/JNK/Bcl-xL axis is a promising target for overcoming radioresistance during CRC treatment.

## Supplementary information

Supplemental information

Original Data File

Reproducibility checklist

## Data Availability

The datasets generated during and/or analysed during the current study are available from the corresponding author on reasonable request.
