# Supplementary material for: TRAF4-mediated ubiquitination-dependent activation of JNK/Bcl-xL drives radioresistance
Source: Cell Death Dis. 2023 Feb 10;14(2):102. doi: 10.1038/s41419-023-05637-y (PMC9918491; doi:10.1038/s41419-023-05637-y)
Supplement: Supplementary file 1 — Supplemental information [file 41419_2023_5637_MOESM1_ESM.pdf]

Supplementary Figure 1

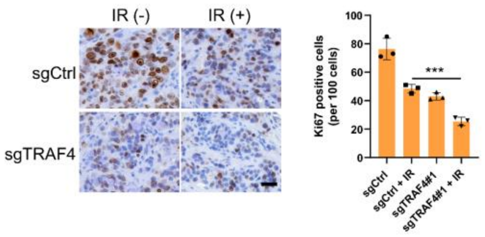

Supplementary Figure 2

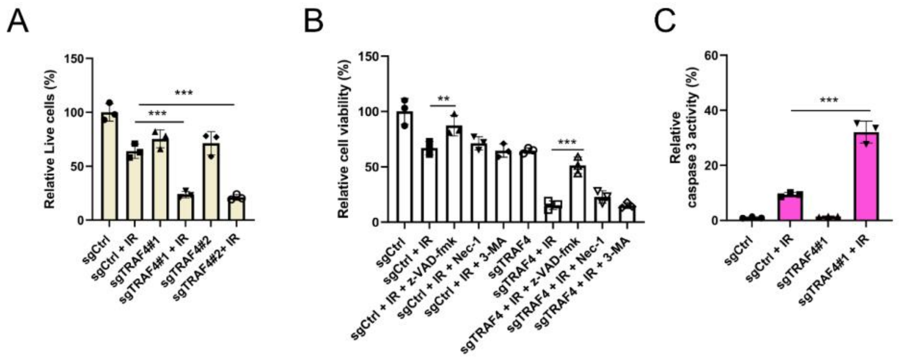

Supplementary Figure 3

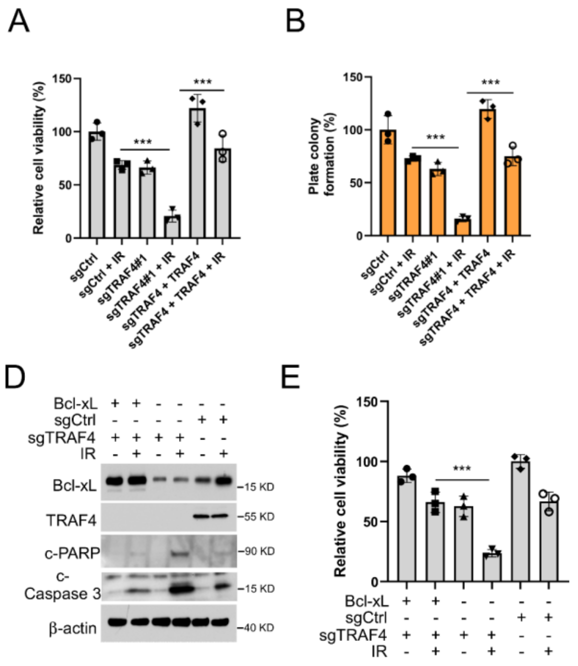

Supplementary Figure 4

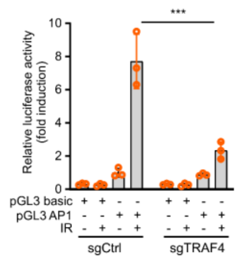

Supplementary Figure 5

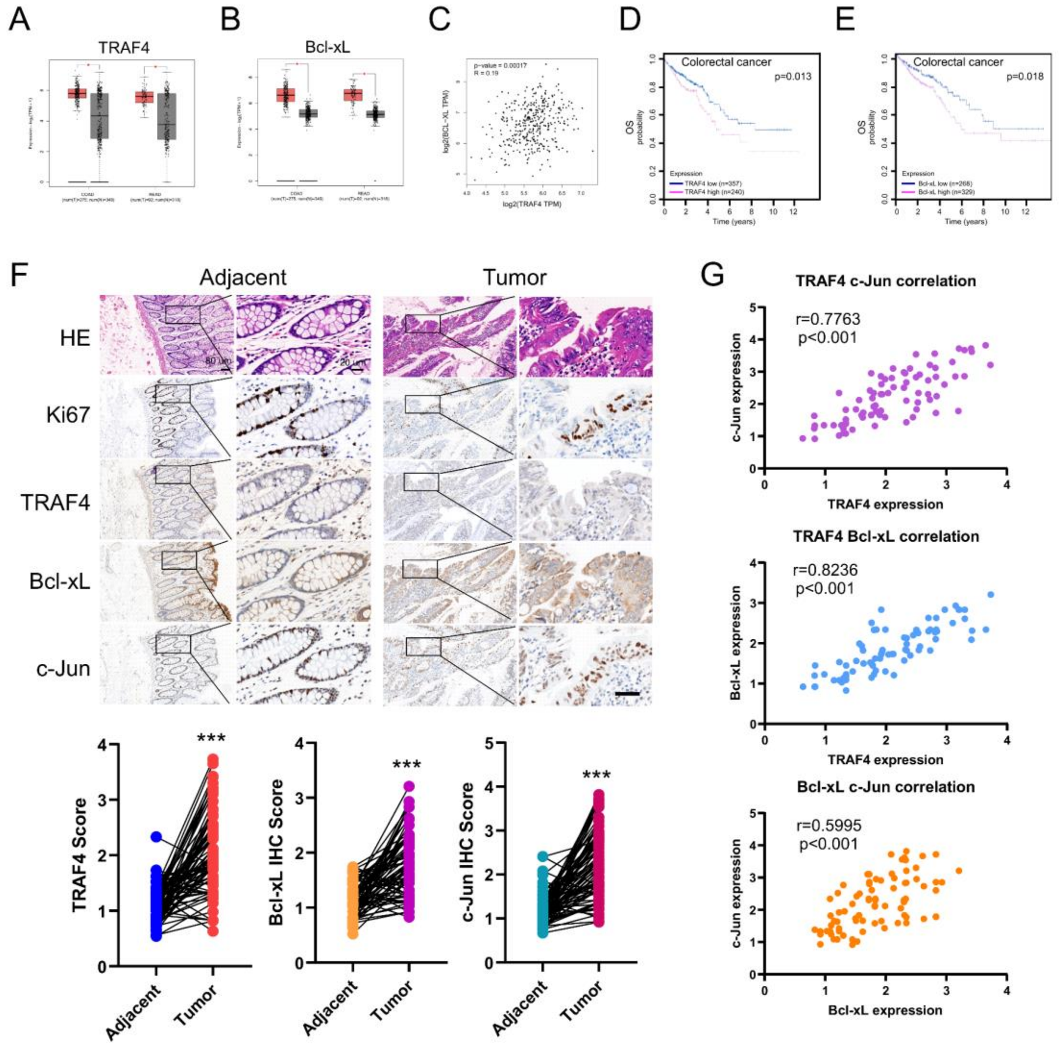

Supplementary Figure 6

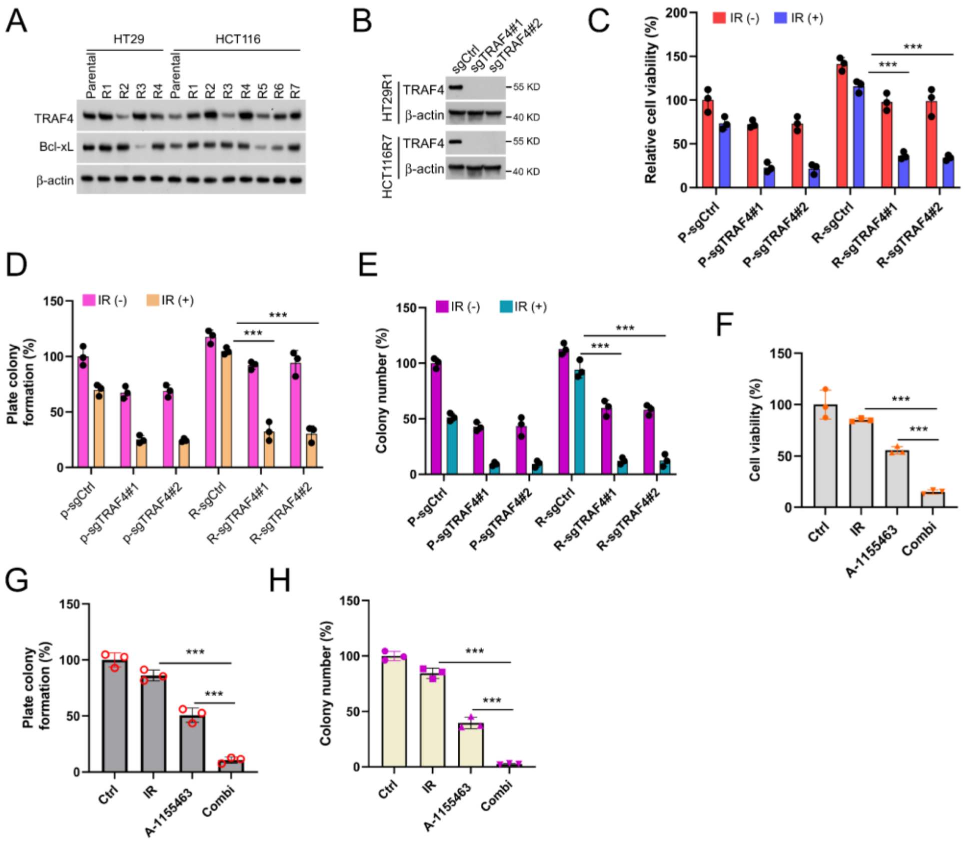

Supplementary Figure 1. Xenograft tumors derived from sg-Ctrl or sg-TRAF4 HCT116 cells were treated with or without IR (2 Gy/twice per week) for 36 days. IHC staining was performed to determine Ki67-positive cells. n=5 mice per group. ***p<0.001. Scale bar, 25 μm.

Supplementary Figure 2. A. HCT116 cells were treated with or without IR (4 Gy) and cultured for 72 h. The population of live cells was determined by trypan blue exclusion assay. ***p<0.001. B. HCT116 cells were pretreated with apoptosis inhibitor z-VAD-fmk, necroptosis inhibitor necrostatin-1, and autophagy inhibitor 3-MA for 4 h, followed by IR (4 Gy) treatment and cultured for 72 h. Cell viability was detected by MTS assay. ***p<0.001. C. HCT116 cells were treated with or without IR (4 Gy) and cultured for 72 h. The Caspase 3 Assay Kit was used to analyze caspase 3 activity. ***p<0.001.

Supplementary Figure 3. A-C. Flag-TRAF4 was transfected into sgTRAF4-HT29 cells for 24 h, followed by IR (4 Gy) treatment, and cultured for 72 h. MTS assay was performed to detect the cell viability (A); Plate colony formation assay was performed to analyze the cell proliferation capacity (B); Soft agar assay was conducted to assess the anchorage-independent cell proliferation ability (C). ***p<0.001. D and E. TRAF4 knockout-HT29 cells were transfected with Bcl-xL for 24 h, then treated with IR (4 Gy) and cultured for 72h. IB was used to examine the protein expression (D); MTS assay was performed to detect cell viability (E). ***p<0.001.

Supplementary Figure 4. HCT116 cells were transfected with pGL3 basic or pGL3 AP1 for 48h and treated with or without IR (4 Gy). Reporter activity was examined 30 min

later. ***$p<0.001$.

Supplementary Figure 5. A and B. The expression levels of TRAF4 (A) and Bcl-xL (B) mRNA in tumor tissues (COAD and READ) (red box) and non-tumor tissues (grey box) were analyzed by GEPIA (http://gepia.cancer-pku.cn/). *$p<0.05$. C. Association between TRAF4 and Bcl-xL mRNA expression was assessed by GEPIA. D and E. Association between TRAF4 (D) or Bcl-xL (E) protein level and overall survival (OS) of CRC patients assessed by Kaplan–Meier survival curves from Proteinatlas database (https://www.proteinatlas.org/). F. Top, IHC staining was performed to determine Ki67, TRAF4, Bcl-xL and c-Jun in 80 cases of primary CRC tumor tissues and matched adjacent tissues. Bottom, Image-Pro-PLUS (v.6) and Image J (NIH) computer software were used to quantify staining intensity. ***$p<0.001$. Scale bar, 50 μm. G. Scatterplot showing the positive correlation between c-Jun and TRAF4 (top), Bcl-xL and TRAF4 (middle), and c-Jun and Bcl-xL (bottom) expression in primary tumor tissues.

Supplementary Figure 6. A. Immunoblotting for TRAF4 and Bcl-xL expression in parental and radioresistant HCT116 and HT29 cells. B. Immunoblotting for TRAF4 expression in TRAF4 knockout-HCT116R7 (termed HCT116R) and -HT29R1 (termed HT29R) cells. C-E. Parental and radioresistant HCT116 cells expressing sg-Ctrl or sg-TRAF4 were treated with/without IR (4 Gy) for 72 h. MTS assay was used to determine the cell viability (C), plate colony formation assay was performed to analyze the cell proliferation capacity (D), soft agar assay was employed to assess the anchorage-independent cell growth (E). ***$p<0.001$. F-H. HCT116R cells were treated with A-1155463 inhibitor (2 μM), IR (4 Gy), or a combination for 72 h. MTS assay was used

to determine the cell viability (F), plate colony formation assay to analyze the cell proliferation capacity (G) and soft agar assay to assess the anchorage-independent cell growth ability (H). ***p<0.001.
