# Supplementary material for: TRAF4-mediated ubiquitination-dependent activation of JNK/Bcl-xL drives radioresistance
Source: Cell Death Dis. 2023 Feb 10;14(2):102. doi: 10.1038/s41419-023-05637-y (PMC9918491; doi:10.1038/s41419-023-05637-y)

Figure S7 a, Full gel for Figure 1B

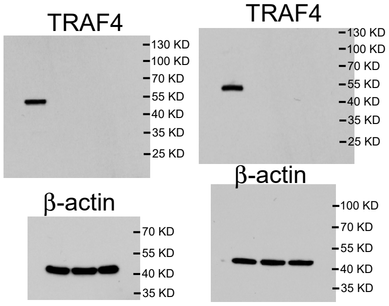

Figure S7 b, Full gel for Figure 2

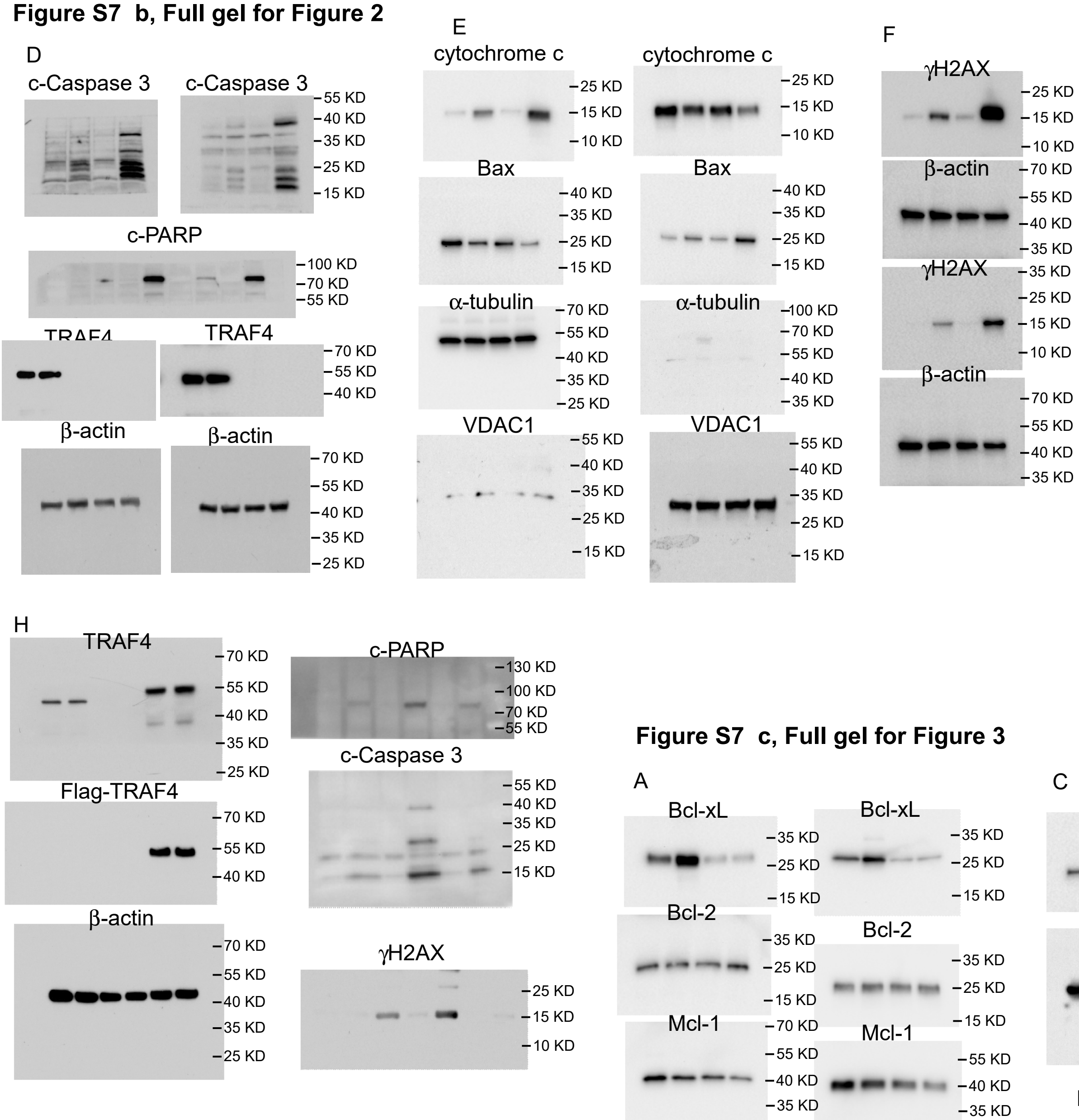

Figure S7 c, Full gel for Figure 3

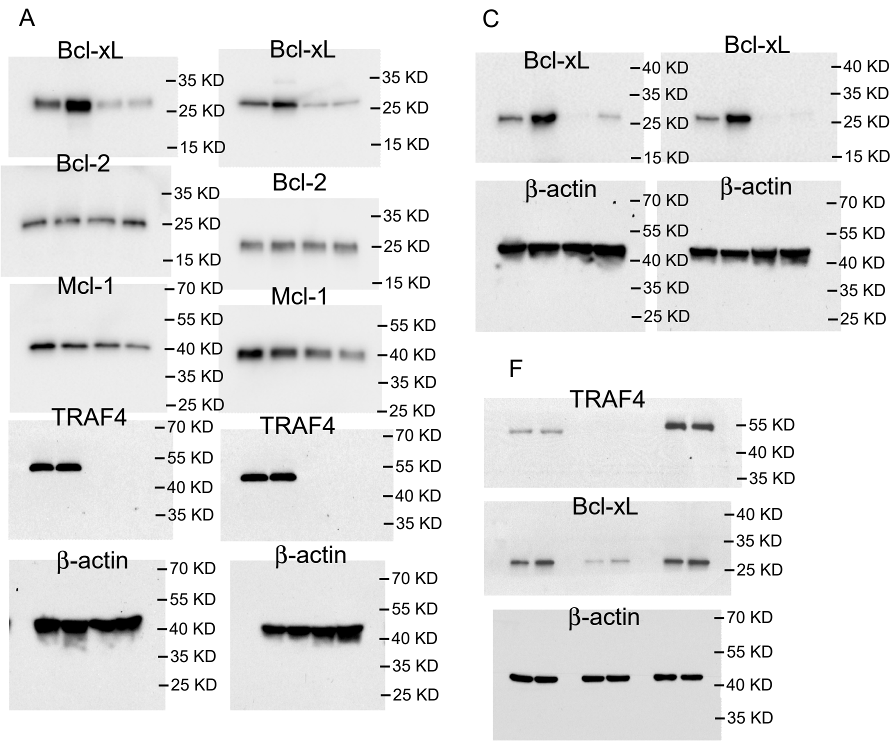

Figure S7 d, Full gel for Figure 4

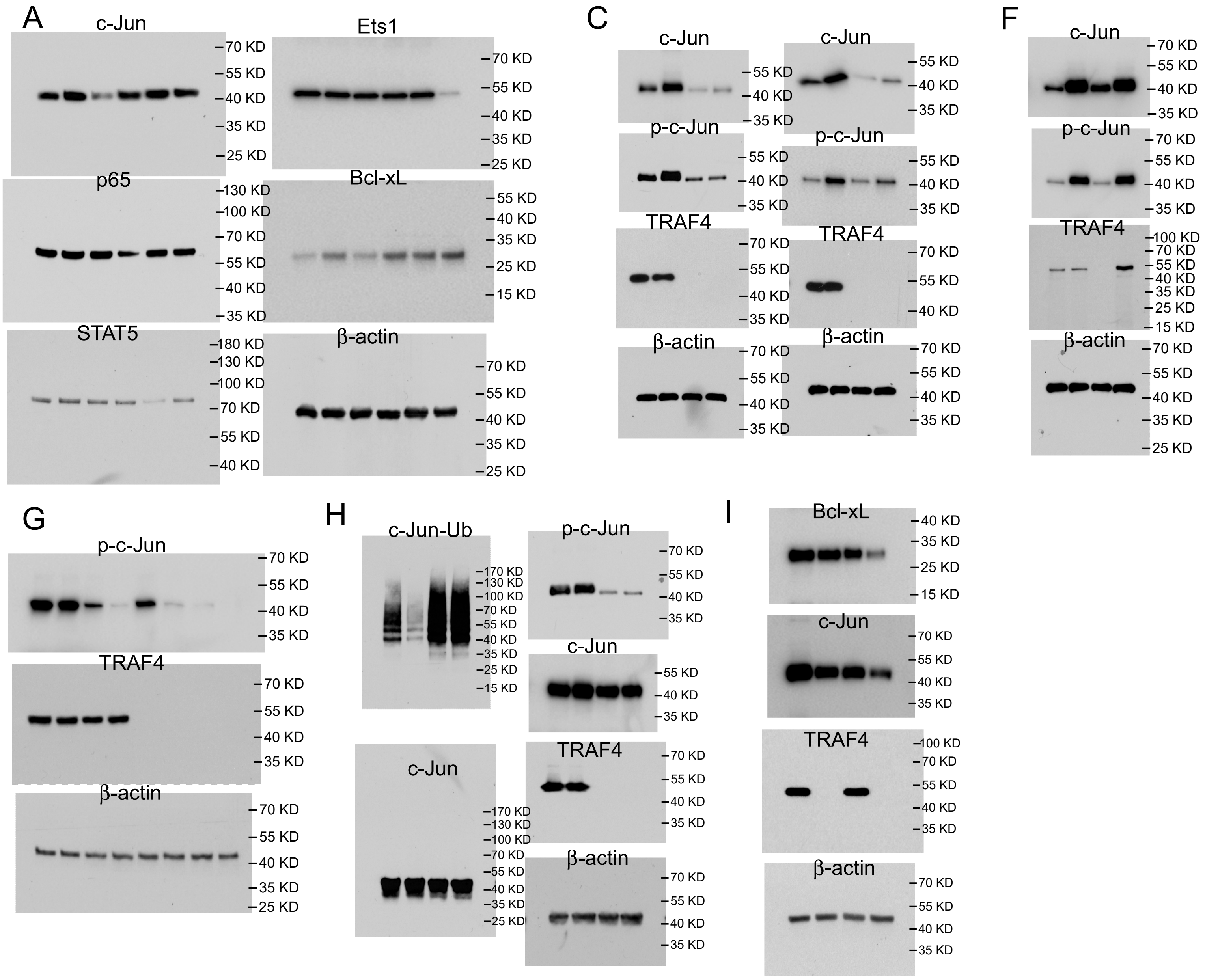

Figure S7 e, Full gel for Figure 5

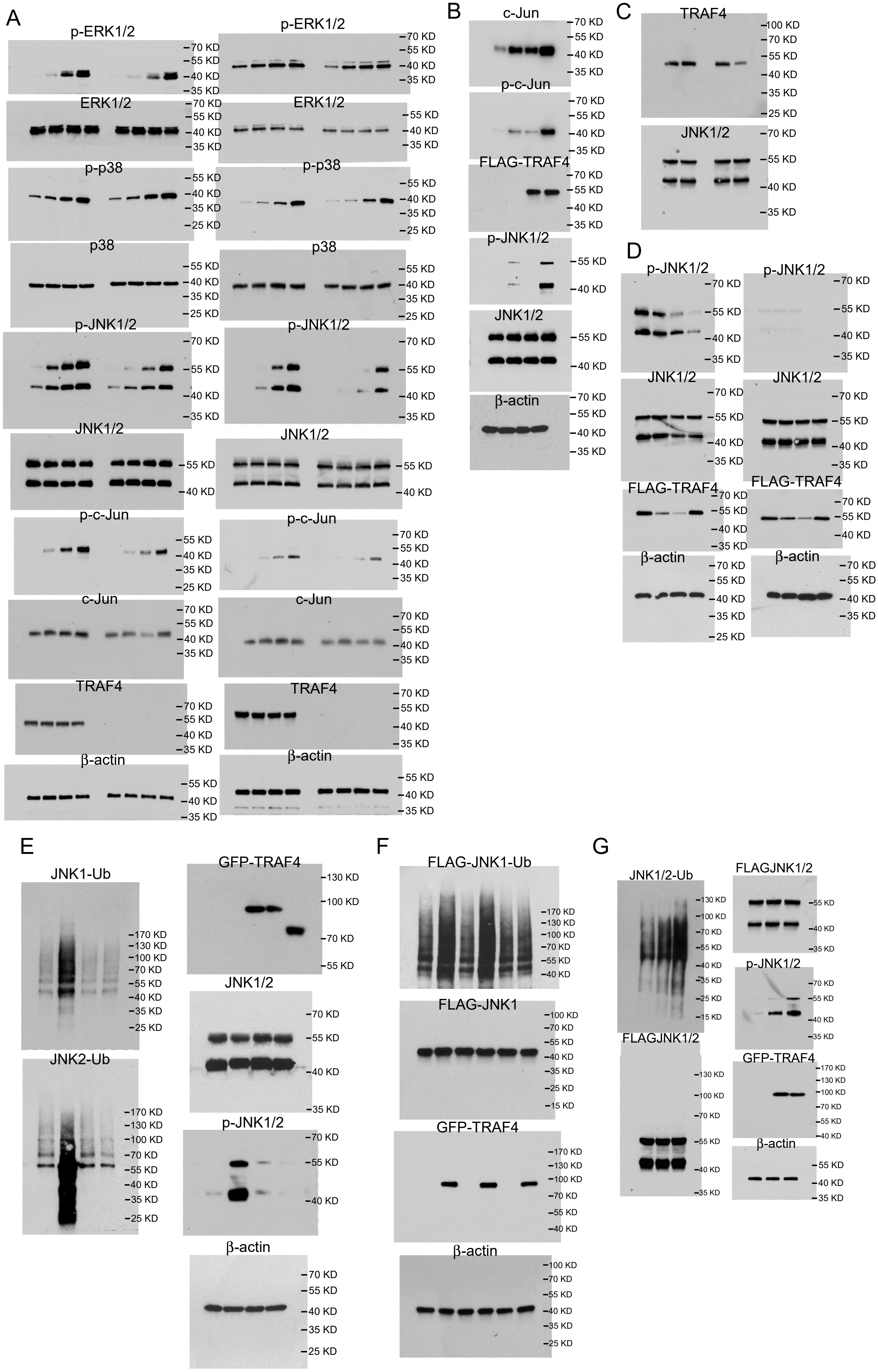

Figure S7 f, Full gel for Figure 8G

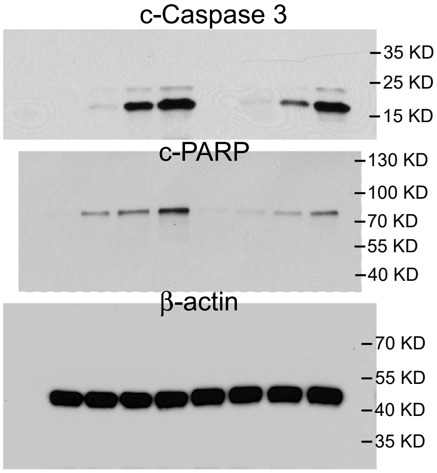

Figure S7 g, Full gel for Supplementary Figure 3D

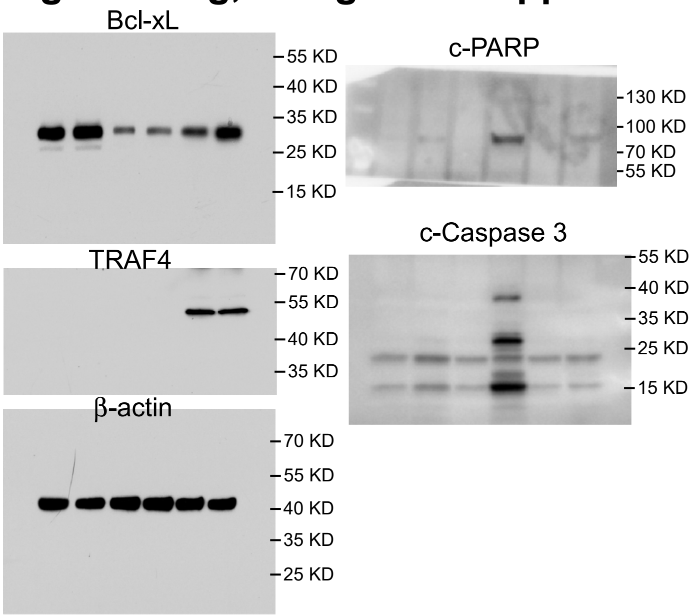

Figure S7 h, Full gel for Supplementary Figure 6

Supplementary Figure 6A

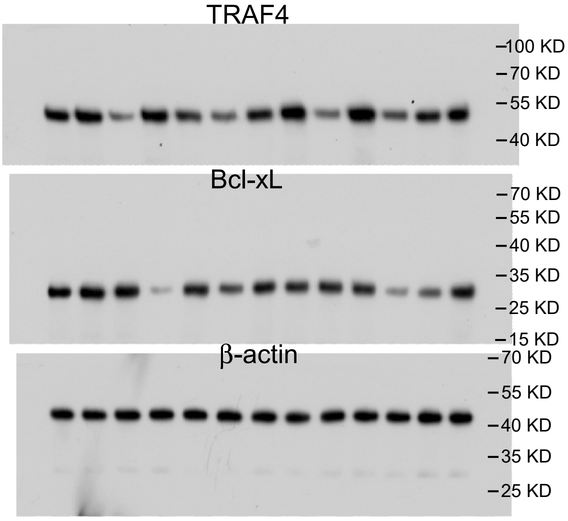

Supplementary Figure 6B

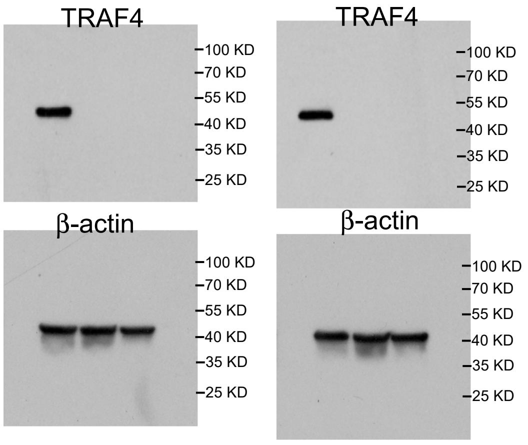

Supplement: Supplementary file 2 — Original Data File [file 41419_2023_5637_MOESM2_ESM.pdf]
